# The association of *TP53* mutations with the resistance of colorectal carcinoma to the insulin-like growth factor-1 receptor inhibitor picropodophyllin

**DOI:** 10.1186/1471-2407-13-521

**Published:** 2013-11-04

**Authors:** Quan Wang, Feng Wei, Guoyue Lv, Chunsheng Li, Tongjun Liu, Costas G Hadjipanayis, Guikai Zhang, Chunhai Hao, Anita C Bellail

**Affiliations:** 1 Department of Gastrointestinal Surgery, Department of Hepatopancreatobiliary Surgery, First Hospital of Jilin University, Changchun, Jilin 130021, China; 2 Department of Colorectal Surgery, Third Hospital of Jilin University, Changchun, Jilin 130033, China; 3 Department of Neurosurgery, Emory University, Atlanta, GA 30322, USA; 4 Department of Pathology & Laboratory Medicine, Emory University, Atlanta, GA 30322, USA

**Keywords:** Apoptosis, Colorectal carcinoma, ERK, IGF-1R, IGF-1R inhibitor, *TP53*

## Abstract

**Background:**

There is growing evidence indicating the insulin-like growth factor 1 receptor (IGF-1R) plays a critical role in the progression of human colorectal carcinomas. IGF-1R is an attractive drug target for the treatment of colon cancer. Picropodophyllin (PPP), of the cyclolignan family, has recently been identified as an IGF-1R inhibitor. The aim of this study is to determine the therapeutic response and mechanism after colorectal carcinoma treatment with PPP.

**Methods:**

Seven colorectal carcinoma cell lines were treated with PPP. Following treatment, cells were analyzed for growth by a cell viability assay, sub-G1 apoptosis by flow cytometry, caspase cleavage and activation of AKT and extracellular signal-regulated kinase (ERK) by western blot analysis. To examine the *in vivo* therapeutic efficacy of PPP, mice implanted with human colorectal carcinoma xenografts underwent PPP treatment.

**Results:**

PPP treatment blocked the phosphorylation of IGF-1R, AKT and ERK and inhibited the growth of *TP53* wild-type but not mutated colorectal carcinoma cell lines. The treatment of PPP also induced apoptosis in *TP53* wild-type cells as evident by the presence of sub-G1 cells and the cleavage of caspase-9, caspase-3, DNA fragmentation factor-45 (DFF45), poly (ADP-ribose) polymerase (PARP), and X-linked inhibitor of apoptosis protein (XIAP). The loss of BAD phosphorylation in the PPP-treated *TP53* wild type cells further suggested that the treatment induced apoptosis through the BAD-mediated mitochondrial pathway. In contrast, PPP treatment failed to induce the phosphorylation of AKT and ERK and caspase cleavage in *TP53* mutated colorectal carcinoma cell lines. Finally, PPP treatment suppressed the growth of xenografts derived from *TP53* wild type but not mutated colorectal carcinoma cells.

**Conclusions:**

We report the association of *TP53* mutations with the resistance of treatment of colorectal carcinoma cells in culture and in a xenograft mouse model with the IGF-1R inhibitor PPP. *TP53* mutations often occur in colorectal carcinomas and could be used as a biomarker to predict the resistance of colorectal carcinomas to the treatment by this IGF-1R inhibitor.

## Background

The IGF-1R signaling pathway plays an important role in the formation and progression of human cancers and has been targeted for cancer treatment [1]. IGF-1R is a membrane- associated receptor tyrosine kinase that controls both cell growth and apoptosis. Insulin-like growth factor-I and -II (IGF-I; IGF-II) ligand binding to IGF-1R leads to the phosphorylation of insulin receptor substrate (IRS) proteins, resulting in the activation of phosphoinositide 3-kinase (PI3K)/AKT and downstream signaling pathways [2]. IGF-1R inhibits the apoptosis pathway through AKT-mediated phosphorylation of BAD, a pro-apoptotic protein of the BCL2 family [3]. Phosphorylated BAD is dissociated from the BCL-2 family proteins that protect mitochondrial membrane potential and thus inhibit mitochondrial release of apoptotic factors [4]. In addition, IGF-1R activates the extracellular signal-regulated kinase (ERK) and nuclear factor-κB (NF-κB) pathway that protect colorectal carcinoma cells from tumor necrosis factor-α (TNFα) induced apoptosis [5]. By activating PI3K/AKT and ERK growth pathways and inhibiting the BAD and TNFα-mediated apoptosis, the IGF-1R signaling pathway promotes the survival, growth, and metastasis of colorectal carcinomas [1,6].

Epidemiological studies have revealed the association of high concentrations of serum IGF-I and IGF-II with the increased risk of developing several human cancers including colorectal carcinomas [7-10]. Examination of colorectal carcinomas has revealed elevation of the transcripts of IGF-I/II [11-13] and IGF-1R [14,15]. These findings suggest that IGF-I/II may interact with IGF-1R on the cancer cell surface and promote cancer growth through paracrine and autocrine loops and targeting of the IGF-IGF-1R pathway may lead to the development of cancer therapeutics [6]. IGF-1R has been targeted by two types of therapeutic agents: IGR-1R neutralizing monoclonal antibodies and small molecule IGF-1R inhibitors [16,17]. Monoclonal antibodies and kinase inhibitors have been characterized in preclinical studies [18] and some have been taken to clinical trials for cancer treatments [19,20]. Preliminary data from current clinical trials have revealed resistance of human cancers to treatment [1,16]. For example, a phase II trial of an IGF-1R antibody has shown a limited response with treatment of metastatic colorectal carcinomas [21].

The characterization of the crystallographic structures of the insulin receptor and IGF-1R has enabled the development of IGF-1R specific inhibitors [22-24]. Picropodophyllin (PPP), a member of the cyclolignan family, has been identified as an IGF-1R inhibitor [25] since it specifically blocks the phosphorylation of the Tyr 1136 residue in the IGF-1R activation loop and thus inhibits the phosphorylation and kinase activity of the receptor [26]. PPP blocks the PI3K/AKT pathway [25], induces apoptosis in multiple myeloma cells [27], and suppresses the growth of multiple myeloma and glioblastoma xenografts [28-30]. Phase I/II trials have been launched for treatment of glioblastoma, hematological malignancies, and non-small cell lung carcinoma by picropodophyllin (AXL1717).

In this study, we investigated the therapeutic response of human colorectal carcinomas with the recently identified IGF-1R inhibitor, PPP [25]. Multiple colorectal carcinoma cell lines were used in addition to colorectal xenografts generated in mice to study the therapeutic response. We examined the IGF-1R downstream AKT and ERK growth pathways and BAD-mediated mitochondrial apoptotic pathway in PPP-treated colorectal carcinoma cells. These studies found the majority of the carcinoma cell lines were resistant to PPP treatment due to the failure of AKT and ERK activation as well as induction of BAD-mediated mitochondrial apoptotic pathways. Furthermore, these studies revealed the association of *TP53* mutations with PPP resistance in the carcinoma cell lines in culture and a xenograft model. While human colorectal carcinomas harbor frequent mutations of *APC*, *TP53*, *PIK3CA* and *KRAS*[31], our findings suggest that the *TP53* mutations are associated with the resistance of colorectal carcinoma to the IGF-1R inhibitor, PPP.

## Methods

### Human colorectal carcinoma cell lines, tumors and normal colon tissues

Human colorectal carcinoma cell lines CACAO-2, COLO-205, COLO-320, DLD-1, HCT-8, HT29 and SW948 were purchased from American Type Collection (ATCC; Rockville, MD). Each cell line was grown in RPMI1640 medium (Invitrogen, Carlsbad, CA) supplemented with 10% fetal bovine serum (FBS). Cells were maintained in a humidified 37°C and 5% CO_2_ incubator. Human colorectal carcinoma and matched adjacent normal colorectal tissue samples were collected in accordance with the protocols approved by the institutional Review Board of the First Hospital of Jilin University. All patients provided written informed consent for the tissue sample collection. This study was approved by the First Hospital Ethical Committee of Jilin University.

### IGF-1R inhibitor and antibodies

PPP were purchased from Calbiochem (EMD Millipore) and dissolved in dimethyl sulfoxide (DSMO) at the concentration of 10 mM and stored in aliquots at −80°C. Recombinant human IGF-I was also purchased from Calbiochem and stored in aliquots at −80°C. The antibodies used in this study were purchased from Cell Signaling Technology (Beverly, MA) against the human caspase-9, phospho-IRS-1, AKT, phospho-AKT (Ser473), ERK, phopho-ERK (Thr202/Thr204), IGF-1R, phospho-IGF-1R (Y1135/1136), BAD and phospho-BAD (Ser112/Ser136). Other primary antibodies used in the study included those against the human poly (ADP-ribose) polymerase (PARP), caspase-3 (StressGen, Ann Harbor, MI), DNF fragmentation factor-45 (DFF45), β-actin, BCL-2 (Santa Cruz Biotechnology, Santa Cruz, CA), MDM2 (sigma Aldrich) and X-linked inhibitor of apoptosis protein (XIAP; Transduction Laboratories, Lexington, KY). The secondary antibodies used in this study were horseradish peroxidase (HRP)-conjugated goat anti-mouse (Southern Biotech, Birmingham, AL) and goat anti-rabbit antibody (Jackson ImmunoResearch Laboratories, West Grove, PA). Protease inhibitor mixture, Triton x-100 and other chemicals were purchased from Sigma-Aldrich. Chemiluminescence was from Amersham Biosciences (Piscataway, NJ).

### Cell viability assay

Cells were grown in 96-well plates at 8x10^3^ cells per well in 100 μl of growth medium. Cells were treated or untreated with PPP in the concentrations as indicated in the Results. After incubation for the times indicated in the Results, cells were washed with a phosphate buffer and 100 μl buffer 0.2 M containing sodium acetate (pH 5.5), 0.1% (v/v) Triton X-100 and 20 mM p-nitrophenyl phosphate was added to each of the wells. The plates were incubated at 37°C for 1.5 hours and the reaction was stopped by the addition of 10 μl 1 M NaOH to each well, Absorbance were measured at 405 nm by a microplate reader (BioRad).

### Flow cytometric assay for the cell cycle and sub-G1 apoptotic cells

Cells were treated with 1 μM PP242 and 2 μM erlotinib, alone or in combination, for 20 hours, harvested, fixed with 70% ethanol, and stained with propidium iodide. The data were acquired using flow cytometry (FACSCanto II Becton Dickinson, Franklin Lakes, NY) and were analyzed using FlowJo software (Tree Star Inc. Ashland, OR). Sub-G1 apoptotic cells were determined as a percentage of the cells.

### Western blotting

Western blotting was performed according to our laboratory protocols [32]. In brief, cells were lysed in a cell lysis buffer (20 nM Tris pH7.4, 150 mM NaCL, 1% NP-40, 10% glycerol,1 mM EGTA, 1 mM EDTA, 5 mM sodium pyrophosphate, 50 mM sodium fluoride, 10 mM β-glycerophosphate, 1 mM sodium vanadate, 0.5 mM DTT, 1 mM PMSF, 2 mM imidazole, 1.15 mM sodium molybdate, 4 mM sodium tartrate dihydrate, and 1x protease inhibitor cocktail). Cell lysates were cleared by centrifugation at 18,000 x g for 15 minutes at 4°C. The supernatant was collected and protein concentrations were determined by the Bradford protein assay following the manufacturer^’^s protocol (Bio-Rad Laboratories). Equal amounts of protein were separated through SDS-PAGE gels and transferred onto nitrocellulose membranes (Bio-Rad Laboratories). The membranes were incubated overnight at 4°C with primary antibody and then for 1 hour with HP-conjugated secondary antibody. The membranes were developed by chemiluminescence.

### Mouse subcutaneous xenografts and treatments

The animal studies were approved by the Institutional Animal Care and Use Committee of Emory University. The HCT-8 cells or Caco2 cells (7 × 10^6^) were implanted subcutaneously into the flank regions of six-week old (about 20 g of body weight) female athymic (nu/nu) mice (Taconic, Hudson, NY). The mice were allowed to develop subcutaneous xenografts and tumor volumes were measured using caliper measurements. When tumors reached approximately 150–200 mm^3^, mice were assigned randomly to 2 experimental groups (n = 4 per group) and treated either with saline as control or PPP (50 mg/kg) through oral gavages, twice per week. Tumor volumes were measured once every 3 days and calculated based on the formula: V =4/3 × π × (length/2 × [width/2]^2^). At the end of treatment, the mice were sacrificed and the tumors were harvested and weighed at necropsy.

### Statistical analysis

All data were presented as means ± SE. Statistical analyses were performed by GraphPad Prism version 5.01 software for Windows (GraphPad Software). The differences in the means between two groups were analyzed with two-tailed unpaired Student’s *t*-test. Results were considered to be statistically significant at P <0.05.

## Results

### *TP53* mutated colorectal carcinoma cells are resistant to PPP treatment

**Figure 1:**
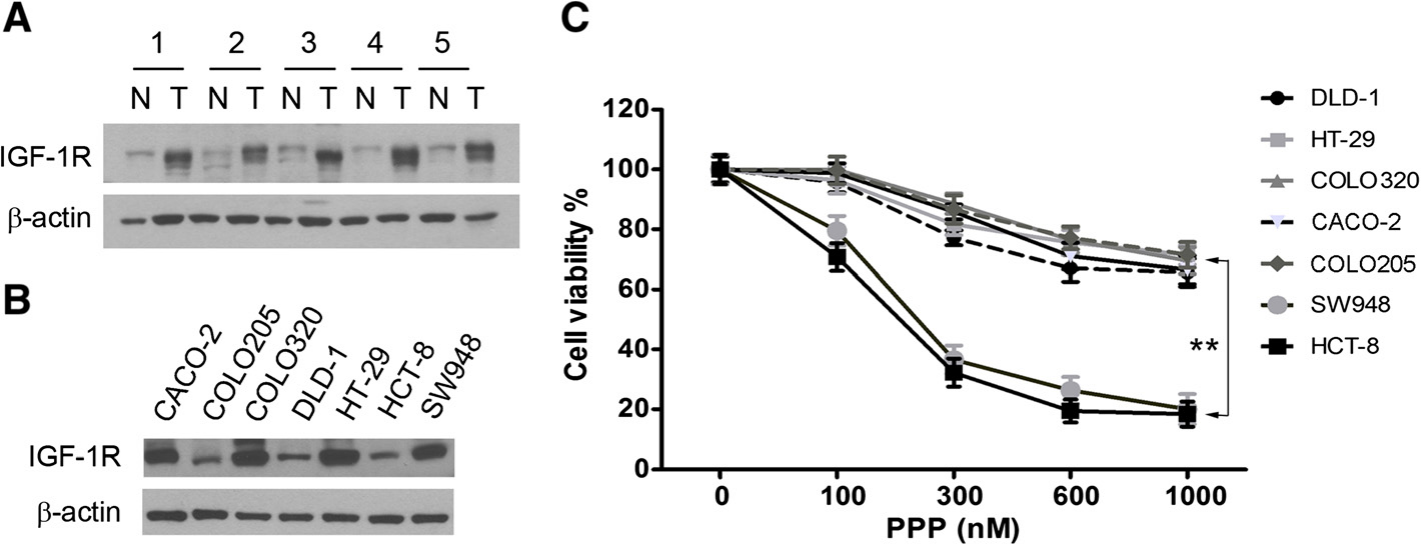
***TP53 *mutation is associated with PPP resistance in colorectal carcinoma cells. (A)**. Western blot analysis of the expression of IGF-1R protein in colorectal carcinoma tumor tissues (T) and matched adjacent normal colorectal tissue (N). β-actin was used as the protein loading control. **(B)**. Western blot detection of IGF-1R protein in a panel of seven colorectal carcinoma cell lines as indicated on the top of the panel. **(C)**. Each of the cell lines was treated with the indicated concentrations of PPP for 72 hours and then analyzed by cell viability assay. The experiment was repeated three times and the data presented as mean + SD (standard deviation). **, p < 0.01.

Earlier studies have revealed increased levels of the IGF-1R mRNA in human colorectal carcinoma tumors [14,15]. To examine the expression of IGF-1R protein, we carried out a western blot analysis of human colorectal carcinoma tumors, together with matched normal colorectal tissue. The results showed that IGF-1R proteins were expressed in the carcinoma tumors at much higher levels than in the matched normal tissue (Figure 1A). We then examined a panel of seven colorectal carcinoma cell lines by western blotting and identified the expression of IGF-1R in each of these cell lines. Nearly half of the cell lines expressed much higher levels of IGF-1R as compared with other cell lines (Figure 1B).

Next, we examined how colorectal carcinoma cell lines respond to PPP treatment. To this end, each of the cell lines was treated with a series of PPP concentrations for 72 hours. A cell viability assay showed PPP treatment significantly inhibited the growth of the sensitive cell lines HCT-8 and SW948. Slight inhibition of the growth of the resistant cell lines CACO-2, COLO-205, COLO-320, DLD-1 and HT-29 was found at much higher doses (Figure 1C). The PPP resistant cell lines were reported with *TP53* mutations [33] according to the Catalogue of Somatic Mutations in Cancer (http://cancer.sanger.ac.uk/cancergenome/projects/cosmic). In contrast, HCT-8 [34] and SW948 (http://cancer.sanger.ac.uk/cancergenome/projects/cosmic) are *TP53* wild-type cell lines. These analyses suggest the association of *TP53* mutations with the PPP resistance of colorectal carcinoma cells to PPP treatment.

### PPP treatment enhances AKT and ERK phosphorylation in *TP53* mt carcinoma cells

**Figure 2:**
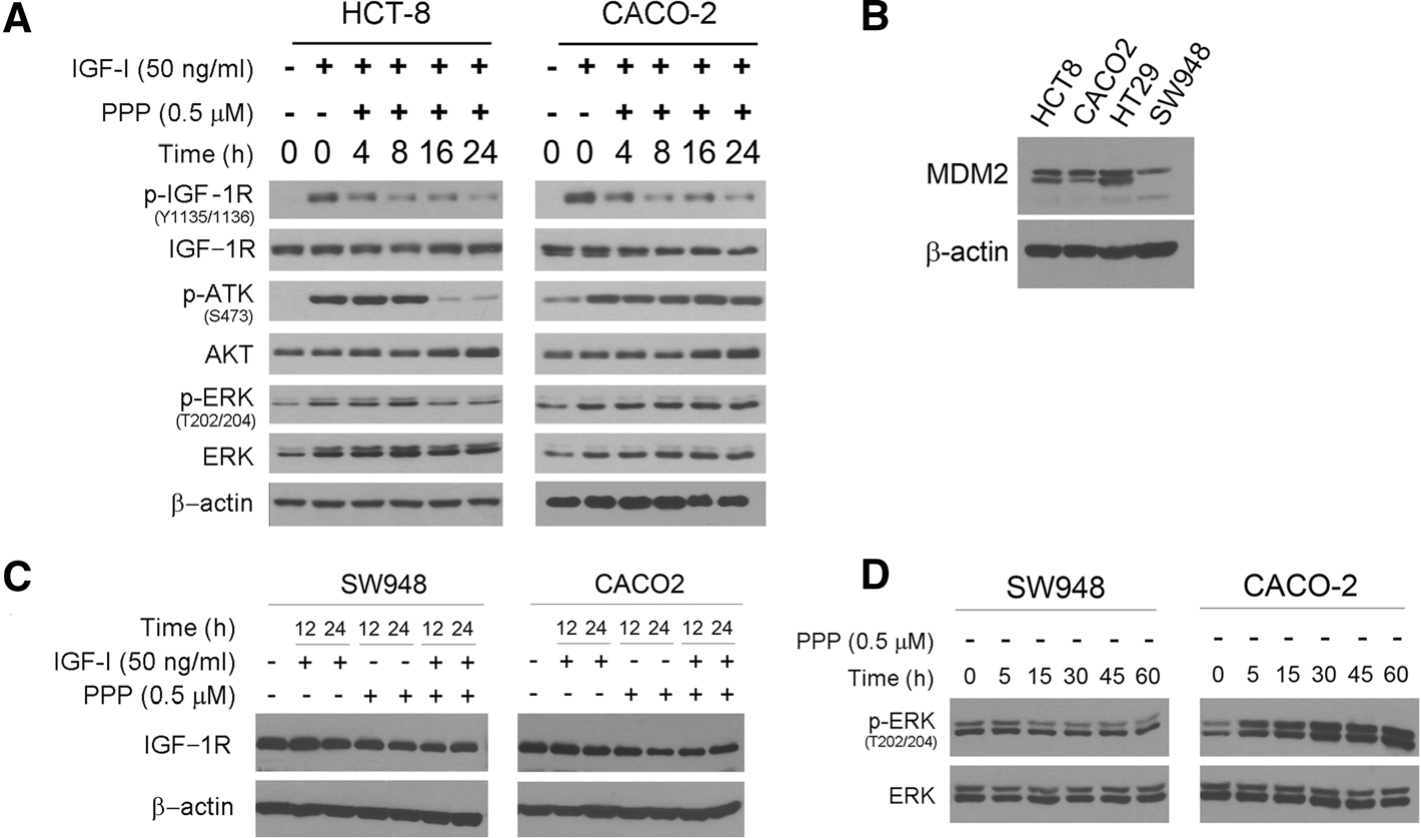
**PPP treatment triggers apoptosis in *TP53 *wild-type but not mutated cells. (A)**. The *TP53* wild-type HCT-8 and mutated CACO-2 cells were treated with 500 nM PPP in the presence or absence of 50 ng/ml IGF-I for the hours as indicated. The treated cells were then examined by western blotting for the presence of the phosphorylated and unphosphorylated IGF-1R, AKT and ERK with β-actin as the loading control. **(B)**. The *TP53* wild-type HCT8 and SW948 and mutated CACO-2 and HT29 were examined by western blotting for the levels of MDM2 protein. **(C)**. The *TP53* wild-type SW948 and mutated CACO-2 cells were treated with 500 nM PPP and 50 ng/ml IGF-I, alone and in combination, for the indicated hours. The cells were then examined by western blotting for the levels of IGF-1R protein. **(D)**. SW948 and CACO-2 cells were treated with 500 nM PPP for the indicated minutes and then analyzed by western blotting for the levels of unphosphorylated and phosphorylated ERK (p-ERK).

To examine the mechanism of PPP resistance, we evaluated whether PPP treatment blocks IGF-1R auto-phosphorylation [26] and inhibits the downstream AKT and ERK pathways [25]. Since IGF-I and IGF-II activate IGF-1R through paracrine and autocrine loops [6], each of the cell lines was therefore treated with 50 ng IGF-I. Western blotting showed that the IGF-I treatment resulted in the phosphorylation of IGF-1R in both the *TP53* wild-type HCT8 and mutated CACO-2 cells (Figure 2A). The cell lines were then treated with 500 nM PPP in the presence of IGF-I and western blotting revealed a decrease in phosphorylation of IGF-1R in a time dependent manner. In contrast, total IGF-1R levels remained unchanged during the treatment. These data indicate that PPP blocks IGF-1R phosphorylation in both *TP53* wild-type and mutated cells. PPP treatment reduced the levels of phosphorylated AKT and ERK in the *TP53* wild-type HCT-8 but not the *TP53* mutated CACO-2 cells; the results suggest that the PPP resistance occurs at IGF-1R downstream intracellular levels in *TP53* mutated cells.

Earlier studies have clearly shown that PPP treatment leads to the downregulation of IGF-1R through MDM2-mediated ubiquitination and degradation of the IGF-1R protein [35]. Both IGF-1R and p53 proteins are the substrates of the ubiquitin ligase MDM2 [36]. To explore the role of MDM2 in the resistance of mutated *TP53* cell lines to PPP, we examined the protein levels of MDM2 in wild-type and mutated *TP53* cell lines by western blotting. The data revealed no difference in the expression of MDM2 protein between *TP5*3 wild-type and mutated cell lines (Figure 2B). Next, we examined the kinetics of IGF-1R degradation under the treatment of IGF-1 and PPP, alone and in combination. To this end, we compared the IGF-1R protein levels between the *TP53* wild-type SW948 and mutated CACO-2 since these two cell lines expressed IGF-1R protein at similar levels (Figure 1B). Western blotting revealed that PPP treatment reduced the levels of IGF-1R protein in both SW948 and CACO-2 cells (Figure 2C) due to the similar expression levels of MDM2 protein between these two cell lines (Figure 2B). These results confirm the earlier reports [35,36] that PPP treatment induces IGF-1R degradation through MDM2-medicated ubiquitination in a p53-independent manner.

MDM2-mediated ubiquitination of IGF-1R with PPP treatment leads to the activation of ERK pathway [37], resulting in the resistance of Ewing’s sarcoma to the treatment of the anti-IGF-1R antibody figitumuab [38]. To explore this mechanism in colorectal carcinoma, we treated SW948 and CACO-2 cell lines with PPP in a dose-dependent manner and found that PPP treatment increased the levels of p-ERK in the *TP53* mutated CACO-2 but not in the *TP53* wild-type SW948 cells (Figure 2D). Taken together, the results suggest that PPP treatment bocks the phosphorylation of IGF-1R and inhibits the downstream ERK pathway in *TP53* wild type colorectal carcinoma cells. In contrast, *TP53* mutated carcinoma cells are resistant to the PPP treatment in part due to its failure of inhibition of the intracellular ERK pathway.

### PPP treatment induces apoptosis in *TP53* wild-type but not mutated carcinoma cells

**Figure 3:**
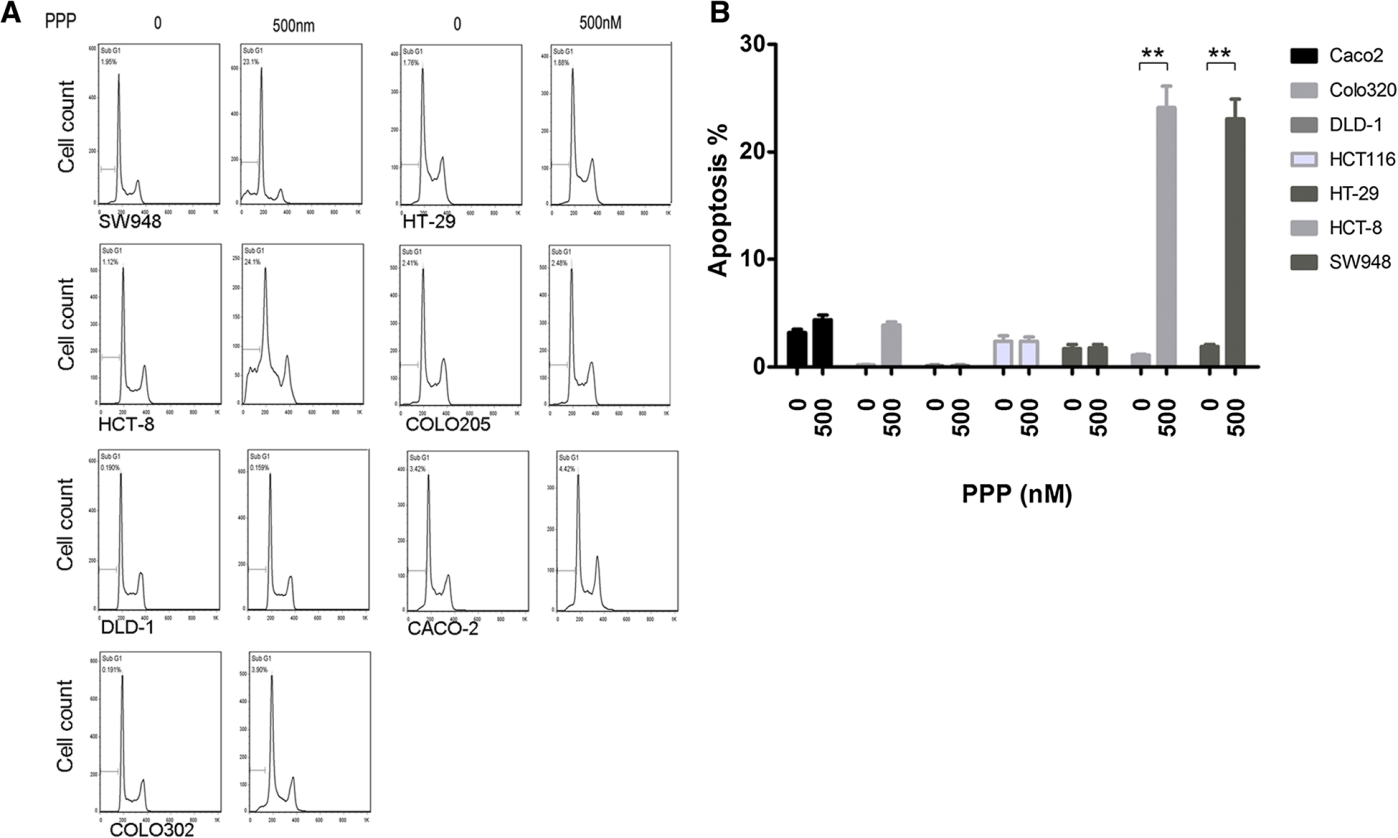
**PPP treatment triggers apoptosis in *TP53 *wild type but not mutated cells. (A)**. Each of the cell lines was treated with 500 nM PPP for 24 hours and subjected to flow cytometry for the detection of the cells in sub-G1 and cell cycle phases. **(B)**. The experiment was repeated three times and the percentage of sub-G1 apoptotic cells is summarized in this histogram as mean + SD. **, p < 0.01.

Earlier studies have shown that PPP treatment inhibits cell growth and induces apoptosis in different types of cancer cells [25,27]. To examine this in colorectal carcinoma cells, we analyzed PPP-treated cells by flow cytometry. The results showed that PPP treatment led to a significant increase of sub-G1 apoptotic cells in the *TP53* wild-type but not mutated cell lines (Figure 3A,B). The results further suggest that *TP53* mutated carcinoma cells are resistant to PPP treatment in part due to its failure of induction of apoptosis in these cells.

**Figure 4:**
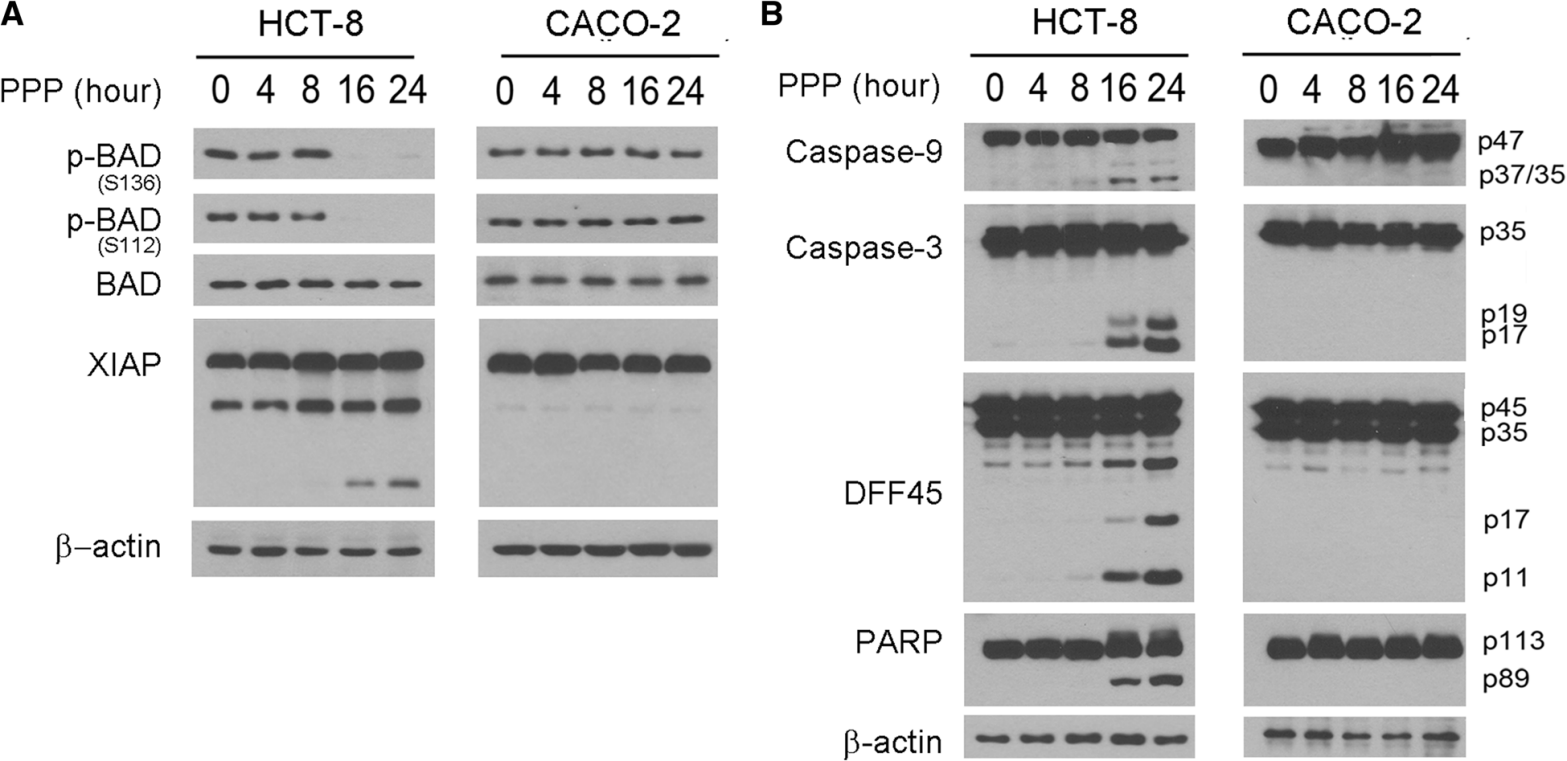
**PPP resistance is in part due to the inhibition of BAD-mediated mitochondrial pathway. (A)**. The *TP53* wild type HCT-8 and mutated CACO-2 were treated with 500 nM PPP for the indicated hours and then subjected to western blotting for the presence of the phosphorylated and unphosphorylated BAD and cleavage of XIAP protein. **(B)**. The PPP treated cells were further examined by western blotting for the cleavage of caspase-9, caspase-3, PARP and DFF45 in HCT-8 and CACO-2 cells with the proteins and cleavage products indicated to the right side of the panel.

IGF-1R activation leads to the inhibition of apoptosis through the AKT/ERK-mediated phosphorylation of BAD [3]. The failure in AKT/ERK activation and apoptosis induction in *TP53* mutated cells under PPP treatment suggests the possibility that BAD phosphorylation may play a role in the PPP resistance. To test this notion, we treated the *TP53* wild type HCT-8 and mutated CACO-2 cells with 500 nM PPP. Lysates from the treated cells were examined by western blot analysis using antibodies against the phosphorylated and unphosphorylated form of BAD. The results showed that the PPP treatment inhibited BAD phosphorylation in *TP53* wild-type but not mutated cells (Figure 4A).

Unphosphorylated BAD interacts with the BCL2 family of proteins and releases their inhibition of the mitochondrial membrane potential [4], leading to the mitochondrial release of apoptosis factors and resulting in caspase-9 activation and initiation of apoptosis through cleavage of the downstream effectors caspase-3, DFF45, and PARP [39]. In addition, the second mitochondria-derived activator of caspase/direct inhibitor of apoptosis binding protein with low pI (SMAC/DIABLO) interacts with THE X-linked inhibitor of apoptosis protein (XIAP), which releases XIAP from binding to caspase-3 and allows caspase-9 cleavage of caspase-3 [40,41]. To examine this mitochondrial pathway in PPP-induced apoptosis, we showed that the treatment of PPP led to the cleavage of XIAP (Figure 4A) and caspase-9, caspase-3, PARP, and DFF45 in the *TP53* wild-type HCT-8 but not the mutated CACO-2 cells (Figure 4B). Collectively, the PPP resistance is in part due to the inhibition of BAD-mediated mitochondrial apoptosis in *TP53* mutated colorectal carcinoma cells.

### PPP treatment inhibits *TP53* wild type but not mutated colorectal carcinoma xenografts

**Figure 5:**
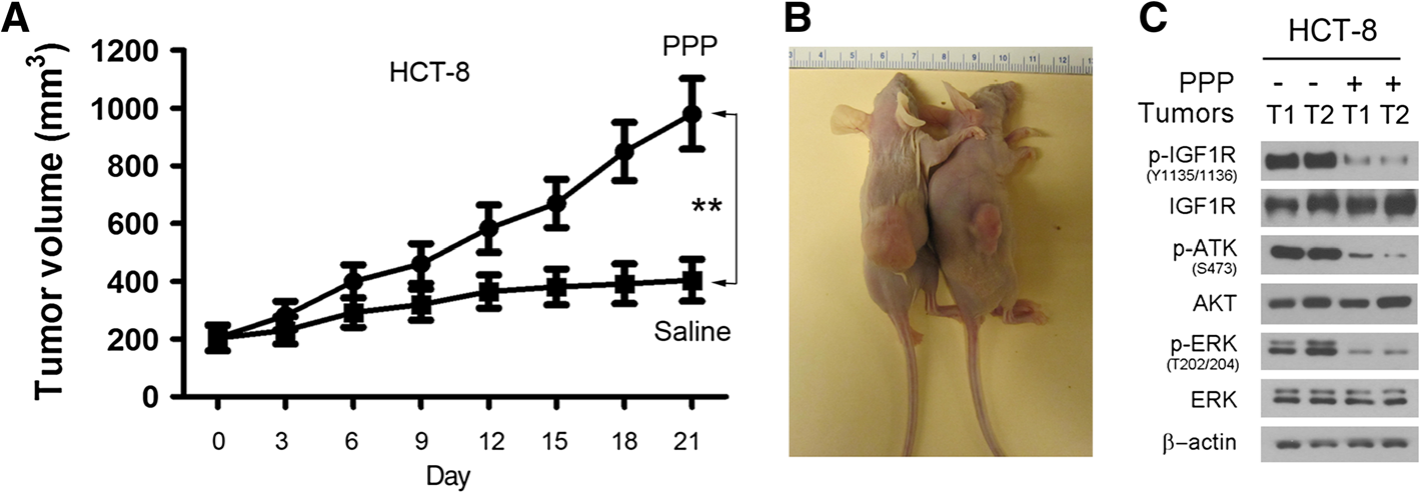
**PPP treatment inhibits the growth of *TP53 *wild type carcinoma xenografts. (A)**. HCT-8 cells were injected subcutaneously in athymic mice for xenograft formation. Once the xenografts were formed, the mice were treated either with PPP (50 mg/kg) in the treatment group or saline in the control group through oral gavage, twice per week. The tumor volumes from the same group of mice were grouped and presented as mean ± SD. **, p < 0.01. **(B)**. The representative mice bearing xenografts were shown at necropsy with saline-treated mouse on the left and PPP-treated mouse on the right. **(C)**. The preventative xenograft tumors (T) from saline treated (−) and PPP treated mice (+) were subjected to western blotting for the presence of the phosphorylated and unphosphorylated IGF-1R, AKT and ERK.

To examine the potential of PPP in treatment of colorectal carcinoma, we first injected the *TP53* wild-type HCT-8 cells subcutaneously in athymic (nu/nu) mice for the generation of subcutaneous flank xenografts. The mice were closely monitored and once xenografts reached approximate size of 150–200 mm^3^, the mice were divided into two groups. In the treatment group, mice were treated with PPP (50 mg/kg) and in the control group, mice were treated with saline. The mice were treated through oral gavage, twice per week for three weeks. Tumor volumes were measured and the results showed that PPP treatment significantly inhibited the growth of the *TP53* wild-type HCT-8 colorectal carcinoma xenografts (Figure 5A). At necropsy, a significant difference in the tumor sizes was observed between the control and treatment mice (Figure 5B). The xenografts were removed and tumor lysates were subjected to western blot analysis. The results showed that PPP treatment inhibited the phosphorylation of IGF-1R, AKT, and ERK in the *TP53* wild-type HCT-8 colorectal carcinoma xenografts (Figure 5C).

**Figure 6:**
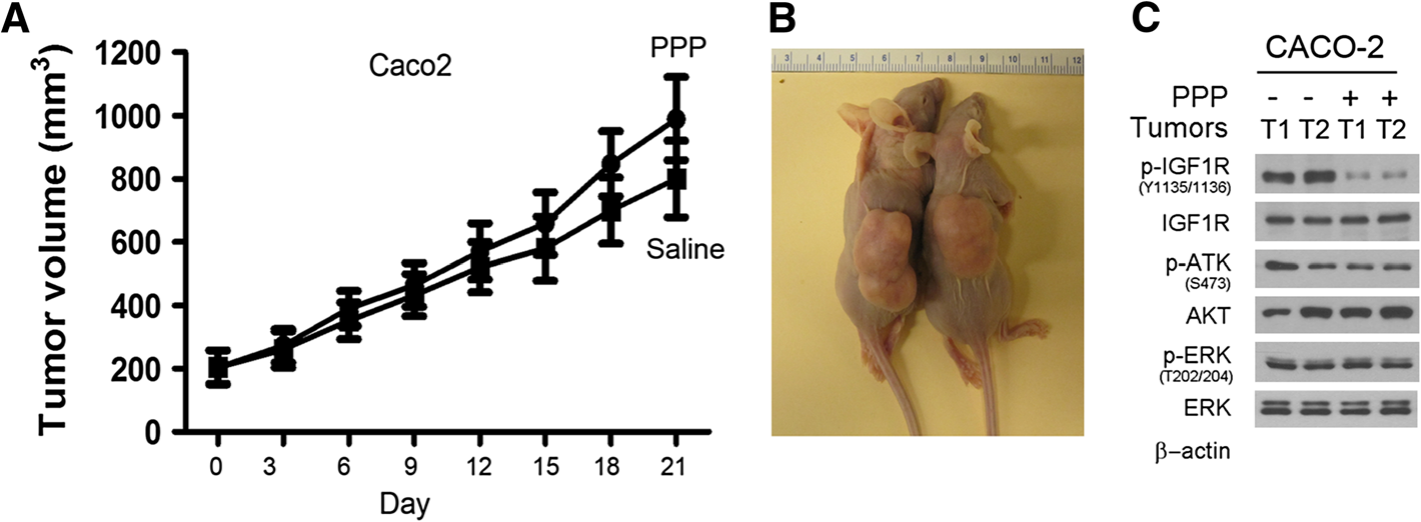
***TP53 *mutated colorectal carcinoma xenografts are resistant to PPP treatment. (A)**. The *TP53* mutated CACO-2 cells were injected subcutaneously in mice and once the subcutaneous xenografts were formed, the mice were treated either with PPP (50 mg/kg) or saline through oral gavage, twice per week. The tumor volumes from the same group of mice were grouped and presented as mean ± SD. **, p < 0.01. **(B)**. The mice bearing xenografts were shown at necropsy with a saline-treated mouse on the left and a PPP-treated mouse on the right. **(C)**. The xenograft tumors (T) from saline treated (−) and PPP treated mice (+) were examined by western blotting for the presence of the phosphorylated and unphosphorylated IGF-1R, AKT and ERK.

To examine whether the *TP53* mutated colorectal carcinoma xenografts resist the treatment of PPP, we injected CACO-2 cells subcutaneously in athymic (nu/nu) mice. Once subcutaneous xenografts were formed approximately 150–200 mm^3^ in size, the mice were treated either with PPP (50 mg/kg) or saline through oral gavage, twice per week for three weeks. The results showed no significant difference in the tumor sizes between the treatment and control group of mice, as indicated by the measured tumor volumes (Figure 6A) and the tumor sizes as observed at necropsy (Figure 6B). Western blot analysis of the representative xenograft tissues showed that PPP treatment failed to inhibit the phosphorylation of IGF-1R, AKT and ERK in the *TP53* mutated CACO-2 colorectal carcinoma xenografts (Figure 6C). Taken together, these studies suggest that *TP53* wild type colorectal carcinoma may respond to the treatment of PPP whereas *TP53* mutated carcinomas are most likely resistant to the treatment.

## Discussion

Colorectal carcinoma is the second leading cause of cancer-related deaths in the United States [42]; thus, there is an urgent need for the development of novel and effective treatment of this devastating human disease. Recent studies have provided several lines of evidence indicating that targeting of IGF-1R may as serve as the basis for clinical treatment of colorectal carcinoma. High concentrations of serum IGF-I/IGF-II are associated with increased risk for developing colorectal carcinoma [7-9] and the IGF-II gene is the single most overexpressed gene in colorectal carcinomas [43]. Furthermore, colorectal carcinomas express high levels of IGF-I/IGF-II [11-13], IGF-1R mRNA [14,15], and IGF-1R protein, as shown in this study. The higher expression levels of IGF-1R are associated with a higher malignant pathologic grade and late stage of colorectal carcinomas [44].

Preclinical studies have shown that the GEO colorectal carcinoma cell line and xenografts respond to the treatment of a dual IGF-1R/insulin receptor kinase inhibitor, PQIP [45]. However, examination of a large panel of colorectal carcinoma cell lines has suggested that the majority of the cell lines are resistant to this dual inhibitor [46]. The combined treatment of the IGF-1R kinase inhibitor, NVP-AFW541 or PQIP with the epidermal growth factor receptor (EGFR) inhibitor erlotinib or tarceva triggers apoptosis and inhibits growth of colorectal carcinoma cell lines [47,48]. A phase II trial, however, has concluded that the IGF-1R neutralizing antibody IMC-A12, alone or in combination with the EGFR antibody cetuximab, is insufficient for the treatment of colorectal carcinomas [21]. Currently, clinical trials of IGF-1R antibodies and kinase inhibitors are ongoing in treating various human cancers. These trails may benefit from studies of the mechanisms in drug resistance and identification of biomarkers that can predict cancer responsiveness to IGF-1R targeted therapies.

After examining a panel of colorectal carcinoma cell lines and xenografts, we have found that the cell lines respond differently to the treatment of PPP, an IGF-1R inhibitor [25]. Some of the cell lines are sensitive whereas other cell lines are resistant to PPP treatment. In the sensitive lines HCT-8 and SW948, PPP treatment blocks IGF-1R phosphorylation and inhibits its downstream AKT and ERK pathway, and suppresses carcinoma cell growth and xenograft progression. In addition, PPP treatment blocks BAD phosphorylation and activates BAD-mediated apoptosis through the mitochondrial pathway. These findings are consistent with other reports that PPP treatment triggers apoptosis in multiple myeloma cells [27] and suppresses the progression of multiple myeloma and glioblastoma xenografts [28-30]. Phase I/II trails of PPP are currently in place for treating patients with glioblastoma, hematological malignancies, and non-small cell lung carcinoma.

The salient feature of this study is that most colorectal carcinoma cell lines are resistant to the treatment of PPP. PPP treatment does block IGF-1R phosphorylation but fails to inhibit the downstream AKT and ERK pathway or induce BAD-mediated mitochondrial apoptosis. These findings are consistent with the clinical trials of IGF-1R targeted agents that have not shown much clinical activity against human cancers [1,16]. Our data suggest that the lack of therapeutic effect is due to the association of PPP resistance with *TP53* mutations in colorectal carcinomas. The p53 tumor suppressor regulates apoptosis in many types of cells and mutations of the *TP53* gene result in the loss of its function in control of apoptosis in cancer cells [49]. *TP53* mutations commonly occur in human colorectal carcinomas [31]. Our study suggests that *TP53* gene status can be used as a biomarker to predict the responsiveness of colorectal carcinomas to the treatment of IGF-1R targeted therapies.

The discovery of PPP as an IGF-1R inhibitor [25] by a research group at the Karolinska Institute has revealed its mechanism of action through inhibition of IGF-1R phosphorylation [26], which induces G2/M-phase accumulation and apoptosis [27]. This group has further shown that PPP treatment down-regulates the IGF-1R protein through MDM2-mediated ubiquitination and degradation [35]. The MDM2-mediated IGF-1R ubiquitination activates the ERK pathway [37] and leads to the cancer resistance to PPP [38]. The data presented in this manuscript have confirmed the action of PPP in inhibition of cell growth and induction of apoptosis in *TP53* wild-type colorectal carcinoma cells. We have also found a correlation between *TP53* mutation and PPP resistance in human colorectal carcinoma cells. Both p53 and IGF-1R proteins are the substrates of MDM2 and the presence of MDM2 in both *TP53* wild-type and mutated carcinoma cells suggests that PPP-induced ERK activation in *TP53* mutated carcinoma cells occurs through a p53-independent manner. The PPP-induced ERK activation contributes in part to the resistance of *TP53* mutated colorectal carcinoma to the IGF-1R inhibitor PPP.

## Conclusions

The IGF-1R inhibitor, PPP, is currently in clinical trials for the treatment of human cancers. We have found the majority of colorectal carcinoma cell lines are resistant to PPP treatment due to failure of activation of the intracellular AKT and ERK growth pathway and induction of the BAD-induced mitochondrial apoptosis pathway. Furthermore, we have found that *TP53* mutations are associated with PPP resistance in colorectal carcinoma and indicated that determining the *TP53* gene status as wild-type or mutated can be used as a biomarker to predict the responsiveness of colorectal carcinoma in human clinical trials.

## Competing interests

The authors declare that they have no competing interests.

## Authors’ contributions

QW and ABC designed the study; QW, FW, CL, KZ and ACB performed the experiments; QW and FW analyzed and interpreted the results; GL, TL and CH contributed materials. ACB and CH wrote the manuscript. CGH edited and revised the manuscript. All authors read and approved the final manuscript.

## Pre-publication history

The pre-publication history for this paper can be accessed here:

http://www.biomedcentral.com/1471-2407/13/521/prepub
